# Baseline Fecal Microbiota in Pediatric Patients With Celiac Disease Is Similar to Controls But Dissimilar After 1 Year on the Gluten-Free Diet

**DOI:** 10.1097/PG9.0000000000000127

**Published:** 2021-10-13

**Authors:** Dory Sample, Janelle Fouhse, Seema King, Hien Q. Huynh, Levinus A. Dieleman, Benjamin P. Willing, Justine Turner

**Affiliations:** From the * Department of Pediatrics, University of Alberta, Edmonton, AB, Canada; † Department of Agricultural, Food and Nutritional Science, University of Alberta, Edmonton, AB, Canada; ‡ Department of Medicine, Community Health Sciences, University of Calgary, Calgary, AB, Canada; § Division of Gastroenterology, Department of Medicine, University of Alberta, Edmonton, AB, Canada

**Keywords:** celiac disease, gluten-free diet, microbiota

## Abstract

The objectives of this pilot study were to examine fecal microbiota composition of pediatric patients with celiac disease (CD) before and after a 1-year gluten-free diet (GFD) and to determine the association with symptoms and anti-tissue transglutaminase (aTTG) antibody.

**Methods:**

Stool samples were obtained from pediatric patients with CD and from healthy controls. Patients were classified by the presence (diarrhea, abdominal pain, weight loss) or absence (asymptomatic, headache, fatigue, etc.) of typical CD gastrointestinal symptoms and by aTTG normalization post-GFD intervention (< 7 U/mL). Fecal microbial composition was measured using 16S ribosomal RNA gene amplicon sequencing of the V3–V4 region.

**Results:**

At diagnosis, 13 of 22 patients with CD had typical gastrointestinal symptoms, the remaining patients having atypical or asymptomatic presentations. After a 1-year GFD, all symptomatic patients improved and 9 of 19 had normalized aTTG. Prior to GFD, no distinct microbial signature was observed between patients and controls (*P* = 0.39). Post-GFD, patients with CD had a unique microbial signature with reductions in known fiber-degrading bacteria, including *Blautia*, *Dorea*, *Lactobacillus*, and *Prevotella* compared with controls. Within the patients with CD, microbial composition was not associated with reported symptom presentation or aTTG normalization.

**Conclusions:**

Pediatric patients with CD only had a unique microbial signature compared with healthy controls when placed on the GFD. These results suggest that pediatric patients with CD may not have a unique fecal microbial signature indicative of inherent dysbiosis, in contrast to that suggested for older patients. In children with CD, diet may play a role in shaping microbial composition more so than disease status.

## Abstract 2

Supplemental Digital Content is available in the text.

**What Is Known**

- Presentation of celiac disease (CD) has changed considerably over time to more non-gastrointestinal (GI) symptoms.
- Studies in adults suggest that CD may be associated with microbial dysbiosis.
- Limited information exists regarding the relationship between the gut-associated microbiota and the GI and non-GI presentations of pediatric CD.

**What Is New**

- No baseline dysbiosis was observed for pediatric patients with CD.
- Symptom presentation was not associated with the gut microbiota in pediatric patients with CD.
- The only observed changes in microbial structure in patients with CD compared with healthy controls occurred 1 year after commencing the gluten-free diet, indicating diet drives the gut-associated microbiota in pediatric CD.

## INTRODUCTION

Celiac disease (CD) is an autoimmune enteropathy triggered by ingested gluten in genetically susceptible individuals and is characterized by gut inflammation and villous atrophy (1,2). This inflammatory process can lead to malabsorption and may present with diarrhea and failure to thrive. Increasingly, children with CD present with nonclassical symptoms or are asymptomatic and identified through screening efforts (2–5). The incidence of CD has risen over the past several decades, especially in children, and is currently reported at approximately 1%–2% of the population (1,6). The only current treatment for CD is a strict, lifelong gluten-free diet (GFD); unfortunately, the diet is difficult to maintain for a variety of reasons, including hidden gluten or cross-contamination (7).

Some evidence suggests that early life environmental factors, such as cesarean section delivery, formula-feeding, gastrointestinal (GI) infection, and/or exposure to antibiotics may increase risk, implicating a potential relationship with the microbiome (8–10). As such, the intestinal and fecal microbiome has become a target of study for examining potentially modifiable differences between healthy children and those with CD, through dietary adjustments.

Research has shown that children with CD can have an altered gut microbiota compared with healthy controls. However, a recent systematic review has revealed that a conclusive microbial signature of these patients has yet to be established (11). Although pediatric patients with CD can have an altered gut microbiota, GFD intervention can also have a strong impact on overall gut microbiota composition (12). Very few studies have characterized gut microbiota composition in patients with CD as it relates to symptom presentation. Adults with classic GI symptoms or anemia have shown to have lower microbial diversity dominated by Proteobacteria from duodenal biopsies compared with patients with atypical symptoms (13). Pediatric patients with CD with abdominal pain were shown to have enriched *Bacillaceae* and *Enterobacteriaceae*, while those experiencing diarrhea had reduced *Clostridium* cluster XIVa and *Akkermansia* and enriched *Bacillaceae* and *Fusobacterium* in fecal samples (14).

Few studies have examined the microbiome in children with CD in relation to the symptom presentation at diagnosis and again after a year on the GFD. We conducted a pilot study, utilizing stored fecal samples, to examine the fecal microbiome in children with CD compared with controls and to determine if any differences were associated with symptom presentation and response to the GFD.

## MATERIALS AND METHODS

### Study Population

All stool samples and population characteristics analyzed for this project were originally collected from patients with CD (January 2013–October 2013) (15) and controls (November 2008–May 2009) (16) recruited into studies at the Stollery Children’s Hospital and the University Hospital, in Edmonton, Alberta, Canada. Both these and the current study were reviewed and approved by a formally constituted review board at the University of Alberta.

All patients with CD had undergone anti-tissue transglutaminase (aTTG) testing and subsequent endoscopy if needed for diagnosis on a gluten-containing diet. Patients with serological diagnosis followed a modified approach to the European Society of Pediatric Gastroenterology, Hepatology and Nutrition criteria as published (15,17). Patients with CD were excluded if they had diabetes or were not consuming dietary gluten, and were classified according to 2 symptom categories at baseline: (1) presence of GI symptoms (diarrhea, bloating, abdominal pain, weight loss) or (2) absence of symptoms or more atypical presentations (headache, fatigue, foggy mind, weight gain).

At 12-month follow-up after commencing the GFD, patients were classified according to the same symptom categories as well as aTTG normalization, defined as aTTG levels of < 7 U/mL. All patients/caregivers received instruction regarding the GFD using a multidisciplinary approach, including a comprehensive instructional session with an expert dietitian, per standard practice at our site (18), to promote diet acceptance. Adherence with the GFD at the 12-month follow-up visit was recorded. Stool samples were collected at baseline and again at the 12-month visit.

Control stool samples were available from a cohort of healthy, asymptomatic siblings of patients with Crohn’s disease, enrolled in a study to explore associations between increased intestinal permeability and small bowel ulceration (16). Participants were excluded for any known or suspected history of inflammatory bowel disease, CD, other small bowel diseases, obstructive symptoms, recent use of nonsteroidal anti-inflammatory medications, ongoing medical treatment of inflammatory conditions using anti-tumor necrosis factors, prednisone, methotrexate, or azathioprine/6-mercaptopurine, and any significant comorbid illness that could adversely affect outcomes related to capsule endoscopy. Fecal calprotectin, C-reactive protein, and aTTG levels were collected. Stool samples from both patients and controls were collected using standard stool collection kits, frozen at home prior to transport to the hospital, and then frozen at –80°C until analysis.

### Fecal DNA Extraction and 16S Sequencing

Genomic DNA was extracted from stool samples using DNeasy PowerSoil kit (Qiagen, Valencia, CA) according to manufacturers’ instructions from approximately 250 mg of frozen feces. DNA concentration was measured using Quant-iT PicoGreen dsDNA Assay Kit (Thermo Fisher Scientific, Waltham, MA) and diluted to 5 ng/uL. The Illumina 16S metagenomic sequencing library preparation protocol was used to prepare amplicon libraries of the V3–V4 region of the 16S gene. The pooled library was paired-end sequenced on an Illumina MiSeq platform (Illumina, Inc., San Diego, CA) using 2 × 300 cycles.

Sequence data were analyzed using a QIIME2 pipeline (Qiime2 v2019.4) (19). Sequences were checked for quality and trimmed if the average quality was below 20, resulting in the forward and reverse reads being truncated at 260 and 220 nt, respectively. The Dada2-plugin was used to denoise the sequence data and generate feature data tables (20). The amplicon sequence variants were aligned with mafft (21) to construct a phylogenetic tree with fasttree2 (22). Taxonomic classification of the bacterial 16S ribosomal RNA gene sequences was made using the Greengenes (13_8 release) reference sequence database (23), with amplicons for the domain of interested extracted using the primer sequences targeting the V3–V4 regions of the 16S ribosomal RNA gene and the q2-feature-classifier extract read method (24).

The R package, Phyloseq, was used to visualize changes to microbial community diversity using Chao1 and Shannon indices and microbial community structure using the Bray-Curtis dissimilarity and principal coordinate analysis (PCoA) and analysis of similarities (ANOSIM) was used to test differences between treatment groups (25). Differential abundance of predominant taxa at the phylum and genus levels were compared between patients with CD and controls using a Wald parametric test in DESeq2 Bioconductor package in R using a false-discovery rate threshold of 0.15 (26).

## RESULTS

### Clinical Characteristics

There were 22 patients with CD (age 4–14 years, mean 8.1 years; 15 female) included with stool available both at diagnosis and after 1 year on a GFD. Most patients (82%) were diagnosed with CD serologically. Baseline aTTG levels ranged from 24 to 1650 U/mL; 2 patients had aTTG levels < 200 U/mL and underwent biopsy for confirmation of disease. Thirteen patients with CD presented with typical GI symptoms, 6 patients had atypical symptoms, and 3 patients were asymptomatic. Patients with atypical symptoms and those who were asymptomatic were combined for analysis purposes. Presentation of symptoms by age group (4–8 and 9–14 years) was not different between typical GI and atypical symptoms/asymptomatic (*P* = 0.93). At 1-year follow-up, all children who reported (18/22) indicated adherence with the GFD. Improvement in symptoms was reported by 16 patients, with the 3 asymptomatic patients maintaining their symptom status (no data on 3 patients). Serum aTTG levels were available at 1-year follow-up for 17 patients; all had improved and levels had normalized for 9 patients.

Control samples were obtained from 17 healthy siblings of patients with inflammatory bowel disease (age 9–19 years, mean 14.3 years; 8 female). All had aTTG levels < 7 U/mL, normal C-reactive protein levels, and 10 of 12 samples were normal for fecal calprotectin.

### Fecal-Associated Microbiota

Fecal microbiota diversity and composition were measured to determine if children diagnosed with CD at baseline (pre-GFD intervention) had a unique fecal microbial signature compared with nonceliac healthy children. A total of 7 819 568 paired-end reads were obtained, averaging 41 000 reads per sample (range, 4100–12 600), and all samples were rarefied to 4100 for downstream statistical analysis.

**FIGURE 1.**
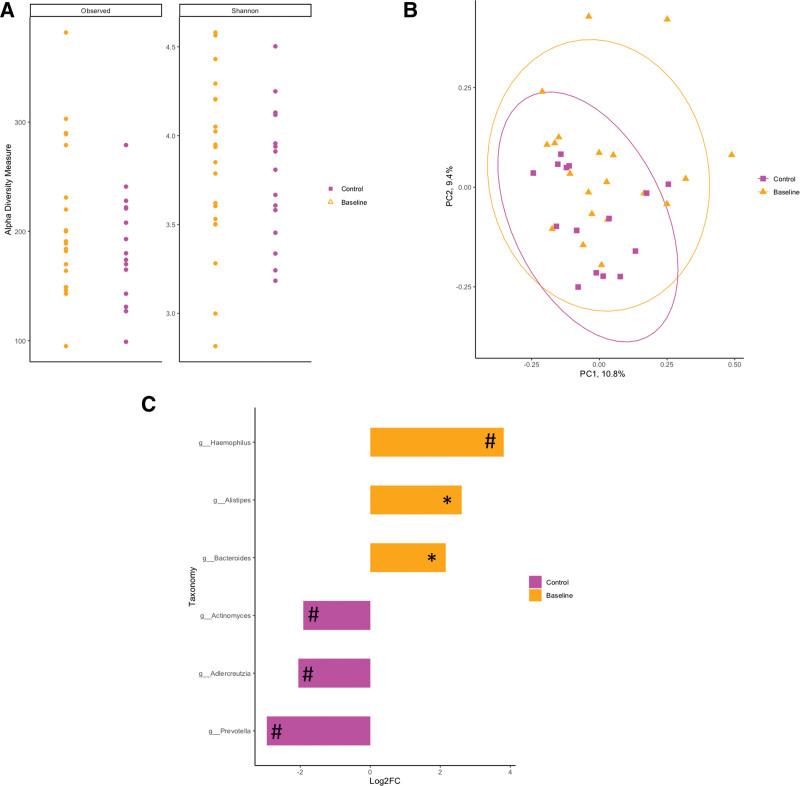
Gut-associated microbial community of diagnosed patients with celiac disease prior to a gluten-free diet intervention did not differ in comparison to healthy nonceliac controls in their diversity (A) observed species (*P* = 0.526); Shannon index (*P* = 0.526); or overall community structure (B) (ANOSIM; *P =* 0.387). C) Taxonomic differences between celiac disease patients and healthy controls were identified by DeSEQ2. Taxa enriched in healthy controls are shown in pink, whereas taxa enriched in patients with celiac disease are displayed in orange (*P < 0.05; #*P* < 0.10). ANOSIM = analysis of similarities; PC = principle coordinate.

Species richness and evenness as measured by observed species and the Shannon index did not differ between controls and patients with CD at baseline (observed species *P* = 0.39; Shannon index *P* = 0.536; Fig. 1A). Furthermore, fecal microbial community structure did not differ between nonceliac healthy controls and baseline patients with CD, as displayed by the indistinct clustering in the PCoA (Fig. 1B; ANOSIM; *P* = 0.387). In patients with CD at baseline, genera *Haemophilus* tended to be enriched (Fig. 1C; *P* < 0.10), whereas genera *Alistipes* and *Bacteroides* were significantly enriched (Fig. 1C; *P* < 0.05). Healthy control patients tended to have enriched genera *Actinomyces*, *Adlercreutzia*, and *Prevotella* (Fig. 1C; *P* < 0.10).

**FIGURE 2.**
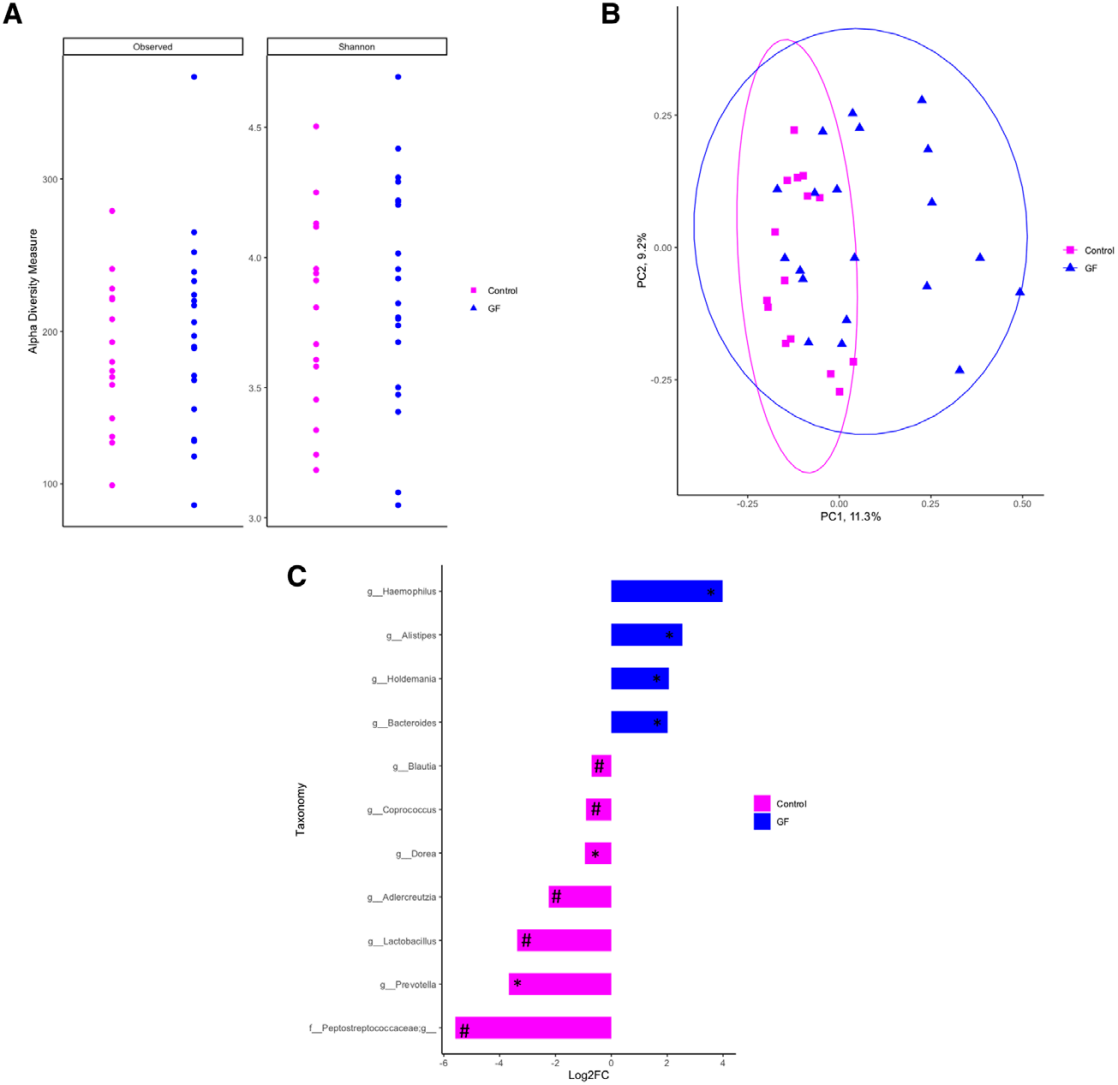
No differences were observed in the diversity of gut-associated microbial communities of healthy controls in comparison to those patients diagnosed with celiac disease after a 1-y GF diet intervention (observed species; *P* = 0.677 and Shannon index; *P* = 0.423) (A). However, microbial community structure tended to be different between patients with celiac disease post-GF diet intervention as displayed by a clustering in the principal coordinate analysis using Bray-Curtis dissimilarity (ANOSIM, *P =* 0.08) (B). C) Taxonomic differences between patients with celiac disease post-GF diet intervention and healthy controls were identified by DeSEQ2. Taxa enriched in healthy controls are shown in pink, whereas taxa enriched in patients with celiac disease after a 1-y GF diet are displayed in blue (*P < 0.05; #*P <* 0.10). ANOSIM = analysis of similarities; GF = gluten-free; PC = principle coordinate.

Fecal microbiota diversity and composition were compared between non-CD healthy controls and patients with CD after a 1-year GFD intervention to determine if dietary intervention causes a shift in microbial structure. Although no differences were observed in species richness or evenness (Fig. 2A; *P* > 0.05), microbial structure tended to differ between patients with CD post-GFD intervention and healthy nonceliac controls, as displayed by the clustering in the PCoA using Bray-Curtis dissimilarity (Fig. 2B; ANOSIM; *P* = 0.08). In post-GFD patients with CD genera enriched included *Haemophilus*, *Alistipes*, *Holdemania*, *Bacteroides*, and *Blautia* (Fig. 2C; *P* < 0.05), whereas *Dorea* and *Prevotella* were enriched (Fig. 2C; *P* < 0.05) in healthy nonceliac controls and *Blautia*, *Coprococcus*, *Adlercreutzia*, *Lactobacillus*, and *Peptostreptococcaceae* tended to be enriched (Fig. 2C; *P* < 0.10).

The fecal microbial composition of patients with CD at baseline and 1-year post-GFD intervention was compared with further understand if GFD intervention influenced microbial composition. Interestingly, no changes to alpha diversity were observed as measured with observed species and Shannon indices (See Figure, Supplemental Digital Content 1, *P* > 0.9, http://links.lww.com/PG9/A59) or overall microbial community structure (See Figure, Supplemental Digital Content 1, *P* > 0.8, http://links.lww.com/PG9/A59).

**FIGURE 3.**
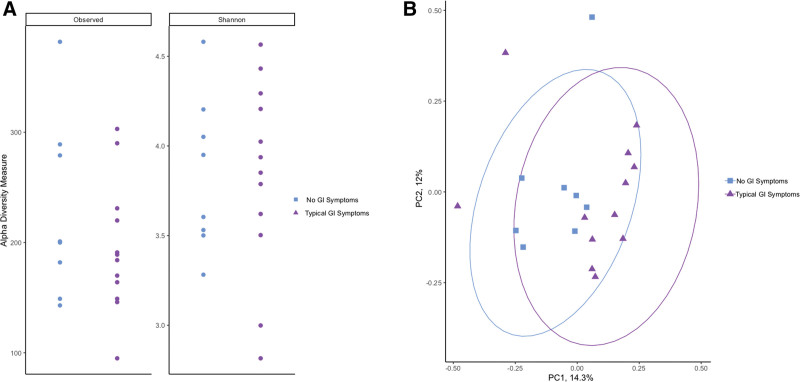
Symptoms of patients with celiac disease at baseline (pre-GFD intervention) (no GI symptoms or typical GI symptoms) were not related to their gut-associated microbial community diversity (observed species; *P* = 0.511 and Shannon index; *P* = 0.877) (A). Patient symptoms did not affect the microbial community structure as displayed by the principal coordinate analysis using Bray-Curtis dissimilarity (ANOSIM; *P =* 0.160) (B). ANOSIM = analysis of similarities; GFD = gluten-free diet; GI = gastrointestinal; PC = principle coordinate.

To determine if there was a specific microbial signature of patients with CD associated with documented symptoms, fecal microbial composition was compared between those patients with CD who were asymptomatic or had atypical symptoms at presentation and those who had typical intestinal symptoms. No microbial signature was observed between these groups, as measured by alpha and beta-diversity (Fig. 3A; observed species, *P* = 0.511; Shannon index, *P* = 0.877; Fig. 3B; beta-diversity ANOSIM, *P* = 0.160). No differences in microbial community structure were observed when comparing patients with CD with Typical GI Symptoms and No GI Symptoms to Controls (See Figure, Supplemental Digital Content 2, ANOSIM *P* = 0.144, http://links.lww.com/PG9/A60). Therefore, to determine if CD had a unique microbial signature regardless of patient symptom, all patients with CD at Baseline were compared against Controls (Refer to Fig. 1A–C).

**FIGURE 4.**
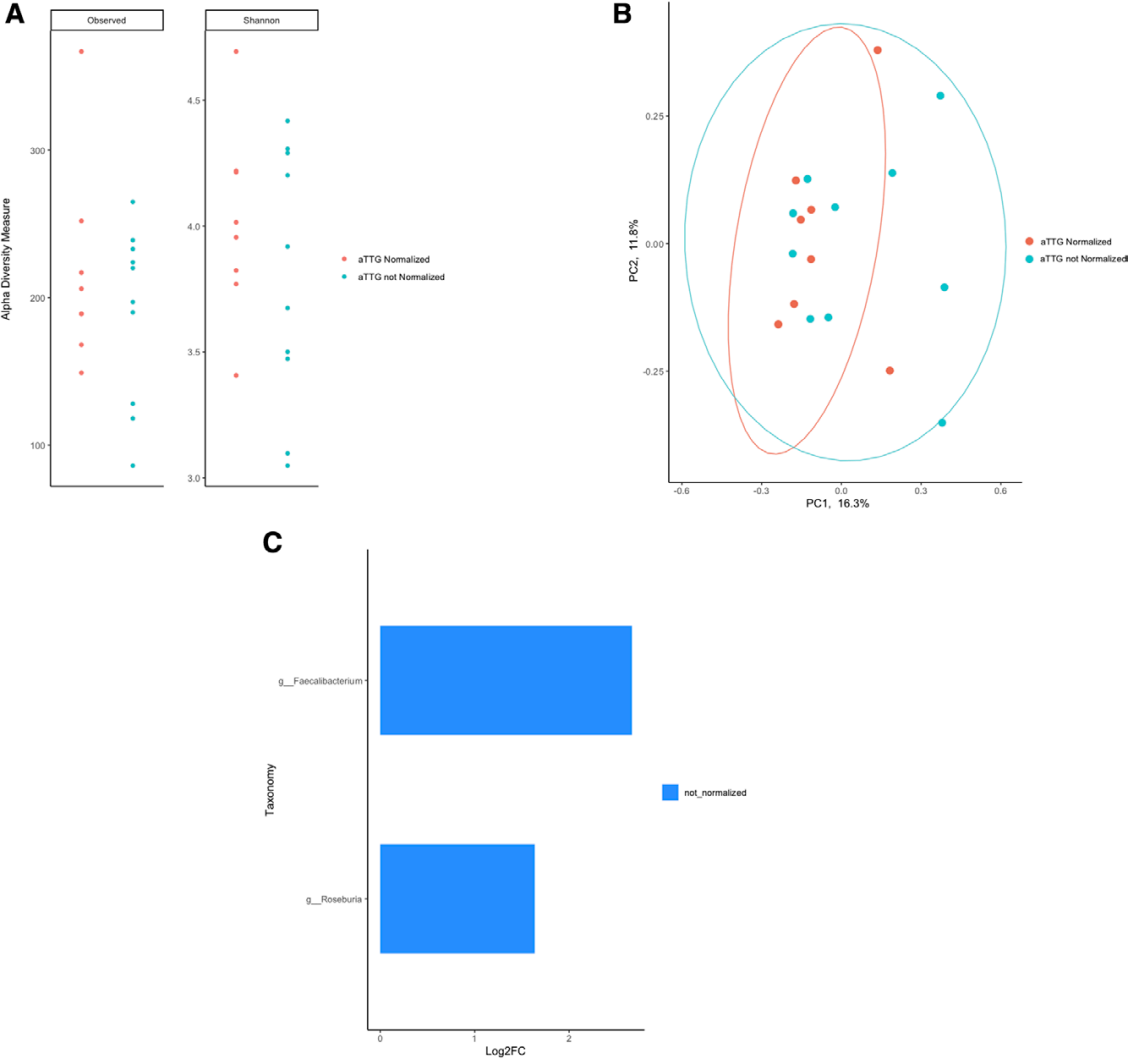
aTTG normalization status in patients with celiac disease after a 1 y GF diet intervention was not related to their gut-associated microbial community diversity (observed species; *P* = 0.859 and Shannon index; *P* = 0.477) (A). Patient aTTG normalization status also did not affect the microbial community structure as displayed by the principal coordinate analysis using Bray-Curtis dissimilarity (ANOSIM; *P* = 0.385) (B). Taxonomy enriched in those patients with not normalized aTTG status post-GF diet intervention is shown in blue (*P <* 0.05) (C). ANOSIM = analysis of similarities; aTTG = anti-tissue transglutaminase; GF = gluten-free; PC = principle coordinate.

No changes in alpha diversity or microbial community structure were observed in patients with CD post-GFD intervention between those patients who had normalized aTTG levels versus those whose aTTG levels did not (Fig. 4A; observed species, *P* = 0.859; Shannon index, *P* = 0.477; and Fig. 4B; beta-diversity ANOSIM, *P* = 0.385). Interestingly, in patients where aTTG levels did not normalize, *Faecalibacterium* and *Roseburia* were enriched (Fig. 4C; *P* < 0.05).

## DISCUSSION

The gut microbiome is an extraordinarily complex ecosystem consisting of trillions of microorganisms. These organisms have several functions, including fiber fermentation, vitamin production, bile acid metabolism, immune system education, and maintenance of gut barrier integrity (27,28). Dysbiosis has been associated with an ever-growing number of disorders, including CD, but it remains unclear if changes in the microbiota are a cause or a consequence of disease. Nonetheless, a better understanding of the impact of dysbiosis, and subsequent development of treatments directed at correcting imbalances, such as probiotics (ie, *Bifidobacteria* and *Lactobacilli* spp.) or prebiotics, may lead to improved health outcomes (29). In the case of CD, the remarkable change in symptom presentation over time is poorly understood and the role of the microbiome in this shift over time warrants exploration. Understanding this relationship might also provide solutions for patients with CD and poor symptom resolution.

Therefore, the objectives of the current pilot study were to characterize how fecal microbial composition in pediatric patients with CD is related to patient-reported symptoms and aTTG status at presentation and follow-up after the GFD intervention, compared with controls. In the current data set, only minor differences in low abundance taxa were observed between pediatric controls and patients with CD at baseline (prior to the GFD intervention) with no changes in overall microbial community composition, which is in agreement with previous research in children (12,30) and adults (31). Interestingly, differences observed in microbial taxa between pediatric patients with CD at baseline and control patients were exaggerated after a 1-year GFD intervention and included increased abundance in the genera *Alistipes*, *Bacteroides*, and *Haemophilus* as well as further reductions in *Prevotella* and *Adlercreutzia*. Others have observed increased abundance of *Haemophilus* in duodenal biopsy samples of untreated patients with CD (30,32) and increased *Bacteroides* in both untreated (33,34) and treated patients with CD (34,35). The fact that *Bacteroides* has been observed to be elevated in both treated and untreated patients with CD may indicate CD has a unique microbial signature. Alternatively, it is plausible that children with CD are already self-selecting low gluten foods prior to diagnosis, explaining minor differences at that time.

In the current study, a GFD diet intervention in patients with CD appears to be the main influencer of fecal-associated microbial community composition. Pediatric patients with CD post-GFD have a unique microbial signature in comparison to controls. Noteworthy changes between patients with CD post-GFD and controls were the characteristic reductions in known carbohydrate degraders including *Blautia*, *Dorea*, *Lactobacillus*, and *Prevotella*. The reduction in carbohydrate degraders in pediatric patients with CD post-GFD is likely due to a reduction in dietary microbial fermentable carbohydrates. It has been documented that the GFD can have a lower content of nondigestible carbohydrates (ie, resistant starch and dietary fiber) when compared with characteristic gluten-containing diets, potentially explaining the reduction in fiber-degrading bacteria (36–42). In pediatric patients with CD, similar findings have been found, revealing that the GFD is a major driver of microbial composition with reductions in carbohydrate utilizing taxonomy observed including *Megamonas*, *Coprococcus*, *Ruminococcus*, *Anaerostipes*, and *Bifidobacteria* (12). The genus *Lactobacillus* has previously been shown to be reduced in pediatric patients with CD on a GFD (35,43–45). In a Danish study with a cross over design, it was found that healthy, nonceliac, subjects consuming minimal gluten diets (~2 g gluten per day) also had reduced fecal abundance of *Blautia* and *Dorea* (46), suggesting that diet, and not CD per se, is the main driver of reducing fiber-degrading bacteria. Interestingly, others have previously seen increased abundance of certain spp. of *Prevotella* in patients with CD. However, it must be noted that this study by Cheng et al (30) compared pediatric patients with CD to healthy control patients who had GI complaints including abdominal pain, gastroesophageal reflux disease, growth retardation, esophagitis, and achalasia. *Prevotella* is a ubiquitous bacterial genus in the human GI tract with different strains having various functional roles in health and disease (47). In alignment with findings from other research groups, our results suggest that a GFD intervention further modifies rather than restoring fecal microbial composition in patients with CD (34,35,45). Furthermore, it has been shown in healthy controls without CD, GFD intervention similarly alters fecal microbial composition, adding to the evidence that diet is the main driver of fecal microbial composition (40,46,48).

Post-GFD, patients with CD with non-normalized aTTG status had elevated levels of *Faecalibacterium* and *Roseburia*. Interestingly, *Faecalibacterium prausnitzii* is elevated in active pediatric patients with CD (untreated) compared with those with nonactive disease (1–2 year GFD) (43). Additionally, abundance of *Roseburia faecis* is increased in healthy subjects on habitual diets versus those on GFD (48). Both *Roseburia* and *Faecalibacterium* are known carbohydrate utilizers and their increased abundance in patients with non-normalized aTTG levels potentially suggests that patients may not have strictly adhered to the GFD recommendations. Normalizing aTTG levels can be an indicator of GFD compliance and it has been shown in a large experimental cohort of pediatric patients with CD (n = 487) that median time to aTTG normalization was 364 days for patients who complied to a GFD (49). In the current study, all patients with CD indicated diet adherence at 1 year, but no objective measure of gluten exposure was available.

It should be noted that the minimal differences in fecal-associated microbial composition in healthy controls and the patients with CD pre-GFD with varying symptoms may be a result of the relatively small sample sizes and high inter-individuality of fecal-microbiota profiles, which can have a strong impact on the results. While the current study found no relationship between gut microbiota composition and reported patient symptoms, other studies have successfully characterized the association between gut microbiota and clinical manifestation of CD. In a comprehensive study following pediatric patients with CD before and after GFD intervention, it was observed that GI severity was positively correlated to abundance of an operational taxonomic unit, classified as *Alistipes* (12). Additional work in pediatric patients with CD also found alterations in microbiota could be associated with GI symptoms (abdominal pain and diarrhea), further suggesting a relationship between GI symptom severity and microbiota exists (14). Patients with CD enrolled in the current study had a large variety of clinical symptoms ranging from typical to atypical, and several patients were asymptomatic. Therefore, profiling fecal microbiota of a larger cohort of patients with CD by specific symptomatic grouping may be of more value in future studies to understand if fecal microbiota is truly a driver or passenger of the disease. This study would have also been strengthened by detailed dietary analysis data before and after the GFD, or, alternatively, a more objective measure of compliance with the GFD like urine or stool peptide testing. Additionally, we studied the fecal microbiome, which may not be representative of the site of inflammation in the gut. Finally, we note that the control subjects in this study were slightly older and included more male subjects. We cannot exclude genetic influences, being siblings of children with Crohn’s disease; however, one advantage of this cohort was the exclusion of CD and of active inflammation.

Microbial composition may play a role in CD pathogenesis due to the ability of microbiota to stimulate the immune system. The observed increase in *Bacteroides* and *Haemophilus* pre- and post-GFD intervention, which has been corroborated by others, suggests this may be a unique microbial signature of CD. However, results of this study are suggestive that diet remains the major driver of fecal-associated microbial composition. This is evidenced by the fact that fecal microbiota in patients with CD post-GFD does not return to that of healthy controls, rather the most compositional changes were observed in patients with CD versus healthy controls post-GFD. Additionally, no changes in microbial community composition were observed when comparing post-GFD patients with CD whose symptoms and aTTG status had normalized versus those who had not yet improved or normalized, furthering the evidence that diet, and not CD itself, is the main driver of microbial composition during active and treated CD.

## ACKNOWLEDGMENTS

B.P.W. and J.T. are co-senior authors, and both conceived of and designed the study. D.S., J.F., S.K., H.Q.H., and L.A.D. acquired the data. D.S., J.F., B.P.W., and J.T. contributed to the data analysis. D.S. and J.F. drafted the article, which all authors reviewed, revised, and approved. All authors agree to be accountable for all aspects of the work.

## Supplementary Material



## References

[1] Ludvigsson JF Murray JA. Epidemiology of celiac disease. Gastroenterol Clin North Am. 2019;48:1–18. 30711202 10.1016/j.gtc.2018.09.004

[2] Hujoel IA Reilly NR Rubio-Tapia A. Celiac disease: clinical features and diagnosis. Gastroenterol Clin North Am. 2019;48:19–37. 30711209 10.1016/j.gtc.2018.09.001

[3] Almallouhi E King KS Patel B. Increasing incidence and altered presentation in a population-based study of pediatric celiac disease in North America. J Pediatr Gastroenterol Nutr. 2017;65:432–437. 28151767 10.1097/MPG.0000000000001532 PMC5538895

[4] Tapsas D Hollén E Stenhammar L. The clinical presentation of coeliac disease in 1030 Swedish children: changing features over the past four decades. Dig Liver Dis. 2016;48:16–22. 26520057 10.1016/j.dld.2015.09.018

[5] Oliveira GN Mohan R Fagbemi A. Review of celiac disease presentation in a pediatric tertiary centre. Arq Gastroenterol. 2018;55:86–93. 29561985 10.1590/S0004-2803.201800000-17

[6] Cichewicz AB Mearns ES Taylor A. Diagnosis and treatment patterns in celiac disease. Dig Dis Sci. 2019;64:2095–2106. 30820708 10.1007/s10620-019-05528-3

[7] Alzaben AS Turner J Shirton L. Assessing nutritional quality and adherence to the gluten-free diet in children and adolescents with celiac disease. Can J Diet Pract Res. 2015;76:56–63. 26067413 10.3148/cjdpr-2014-040

[8] Girbovan A Sur G Samasca G. Dysbiosis a risk factor for celiac disease. Med Microbiol Immunol. 2017;206:83–91. 28204873 10.1007/s00430-017-0496-z

[9] Cristofori F Indrio F Miniello VL. Probiotics in celiac disease. Nutrients. 2018;10:E1824. 10.3390/nu10121824 PMC6316269 30477107

[10] Serena G Lima R Fasano A. Genetic and environmental contributors for celiac disease. Curr Allergy Asthma Rep. 2019;19:40. 31321608 10.1007/s11882-019-0871-5

[11] Abdukhakimova D Dossybayeva K Poddighe D. Fecal and duodenal microbiota in pediatric celiac disease. Front Pediatr. 2021;9:652208. 33968854 10.3389/fped.2021.652208 PMC8100229

[12] Zafeiropoulou K Nichols B Mackinder M. Alterations in intestinal microbiota of children with celiac disease at the time of diagnosis and on a Gluten-free diet. Gastroenterology. 2020;159:2039–2051.e20. 32791131 10.1053/j.gastro.2020.08.007 PMC7773982

[13] Wacklin P Kaukinen K Tuovinen E. The duodenal microbiota composition of adult celiac disease patients is associated with the clinical manifestation of the disease. Inflamm Bowel Dis. 2013;19:934–941. 23478804 10.1097/MIB.0b013e31828029a9

[14] Di Biase AR Marasco G Ravaioli F. Gut microbiota signatures and clinical manifestations in celiac disease children at onset: a pilot study. J Gastroenterol Hepatol. 2021;36:446–454. 32666516 10.1111/jgh.15183

[15] Rajani S Huynh HQ Shirton L. A Canadian study toward changing local practice in the diagnosis of pediatric celiac disease. Can J Gastroenterol Hepatol. 2016;6234160:1–7. 10.1155/2016/6234160 PMC4904635 27446854

[16] Teshima CW Goodman KJ El-Kalla M. Increased intestinal permeability in relatives of patients with Crohn’s disease is not associated with small bowel ulcerations. Clin Gastroenterol Hepatol. 2017;15:1413–1418.e1. 28286191 10.1016/j.cgh.2017.02.028

[17] Saginur M Fawaz AM Spady DW. Antitissue transglutaminase antibody determination versus upper endoscopic biopsy diagnosis of paediatric celiac disease. Paediatr Child Heal. 2013;18:246–250.

[18] Rajani S Sawyer-Bennett J Shirton L. Patient and parent satisfaction with a dietitian- and nurse- led celiac disease clinic for children at the Stollery Children’s Hospital, Edmonton, Alberta. Can J Gastroenterol. 2013;27:463–466. 23936876 10.1155/2013/537160 PMC3956026

[19] Bolyen E Rideout JR Dillon MR. Reproducible, interactive, scalable and extensible microbiome data science using QIIME 2. Nat Biotechnol. 2019;37:548–857. 10.1038/s41587-019-0209-9 PMC7015180 31341288

[20] Callahan BJ McMurdie PJ Rosen MJ. DADA2: high-resolution sample inference from Illumina amplicon data. Nat Methods. 2016;13:581–583. 27214047 10.1038/nmeth.3869 PMC4927377

[21] Katoh K Misawa K Kuma K. MAFFT: a novel method for rapid multiple sequence alignment based on fast Fourier transform. Nucleic Acids Res. 2002;30:3059–3066. 12136088 10.1093/nar/gkf436 PMC135756

[22] Price MN Dehal PS Arkin AP. FastTree 2–approximately maximum-likelihood trees for large alignments. PLoS One. 2010;5:e9490. 20224823 10.1371/journal.pone.0009490 PMC2835736

[23] McDonald D Price MN Goodrich J. An improved Greengenes taxonomy with explicit ranks for ecological and evolutionary analyses of bacteria and archaea. ISME J. 2012;6:610–618. 22134646 10.1038/ismej.2011.139 PMC3280142

[24] Bokulich NA Kaehler BD Rideout JR. Optimizing taxonomic classification of marker-gene amplicon sequences with QIIME 2’s q2-feature-classifier plugin. Microbiome. 2018;6:90. 29773078 10.1186/s40168-018-0470-z PMC5956843

[25] McMurdie PJ Holmes S. phyloseq: an R package for reproducible interactive analysis and graphics of microbiome census data. PLoS One. 2013;8:e61217. 23630581 10.1371/journal.pone.0061217 PMC3632530

[26] Love MI Huber W Anders S. Moderated estimation of fold change and dispersion for RNA-seq data with DESeq2. Genome Biol. 2014;15:550. 25516281 10.1186/s13059-014-0550-8 PMC4302049

[27] Avelar Rodriguez D Peña Vélez R Toro Monjaraz EM. The gut microbiota: a clinically impactful factor in patient health and disease. SN Compr Clin Med. 2019;1:188–199.

[28] Krishnareddy S. The microbiome in celiac disease. Gastroenterol Clin North Am. 2019;48:115–126. 30711204 10.1016/j.gtc.2018.09.008

[29] Marasco G Cirota GG Rossini B. Probiotics, prebiotics and other dietary supplements for gut microbiota modulation in celiac disease patients. Nutrients. 2020;12:E2674. 10.3390/nu12092674 PMC7551848 32887325

[30] Cheng J Kalliomäki M Heilig HG. Duodenal microbiota composition and mucosal homeostasis in pediatric celiac disease. BMC Gastroenterol. 2013;13:113. 23844808 10.1186/1471-230X-13-113 PMC3716955

[31] Bodkhe R Shetty SA Dhotre DP. Comparison of small gut and whole gut microbiota of first-degree relatives with adult celiac disease patients and controls. Front Microbiol. 2019;10:164. 30800106 10.3389/fmicb.2019.00164 PMC6376745

[32] Ou G Hedberg M Hörstedt P. Proximal small intestinal microbiota and identification of rod-shaped bacteria associated with childhood celiac disease. Am J Gastroenterol. 2009;104:3058–3067. 19755974 10.1038/ajg.2009.524

[33] Schippa S Iebba V Barbato M. A distinctive ‘microbial signature’ in celiac pediatric patients. BMC Microbiol. 2010;10:175. 20565734 10.1186/1471-2180-10-175 PMC2906462

[34] Collado MC Donat E Ribes-Koninckx C. Specific duodenal and faecal bacterial groups associated with paediatric coeliac disease. J Clin Pathol. 2009;62:264–269. 18996905 10.1136/jcp.2008.061366

[35] Di Cagno R De Angelis M De Pasquale I. Duodenal and faecal microbiota of celiac children: molecular, phenotype and metabolome characterization. BMC Microbiol. 2011;11:219. 21970810 10.1186/1471-2180-11-219 PMC3206437

[36] Miranda J Lasa A Bustamante MA. Nutritional differences between a gluten-free diet and a diet containing equivalent products with gluten. Plant Foods Hum Nutr. 2014;69:182–187. 24578088 10.1007/s11130-014-0410-4

[37] Kinsey L Burden ST Bannerman E. A dietary survey to determine if patients with coeliac disease are meeting current healthy eating guidelines and how their diet compares to that of the British general population. Eur J Clin Nutr. 2008;62:1333–1342. 17700651 10.1038/sj.ejcn.1602856

[38] Wild D Robins GG Burley VJ. Evidence of high sugar intake, and low fibre and mineral intake, in the gluten-free diet. Aliment Pharmacol Ther. 2010;32:573–581. 20528829 10.1111/j.1365-2036.2010.04386.x

[39] Sanz Y. Effects of a gluten-free diet on gut microbiota and immune function in healthy adult humans. Gut Microbes. 2010;1:135–137. 21327021 10.4161/gmic.1.3.11868 PMC3023594

[40] De Palma G Nadal I Collado MC. Effects of a gluten-free diet on gut microbiota and immune function in healthy adult human subjects. Br J Nutr. 2009;102:1154–1160. 19445821 10.1017/S0007114509371767

[41] Caminero A Meisel M Jabri B. Mechanisms by which gut microorganisms influence food sensitivities. Nat Rev Gastroenterol Hepatol. 2019;16:7–18. 30214038 10.1038/s41575-018-0064-z PMC6767923

[42] Caminero A Verdu EF. Celiac disease: should we care about microbes? Am J Physiol Gastrointest Liver Physiol. 2019;317:G161–G170. 31188640 10.1152/ajpgi.00099.2019 PMC6734371

[43] Nadal I Donant E Ribes-Koninckx C. Imbalance in the composition of the duodenal microbiota of children with coeliac disease. J Med Microbiol. 2007;56(pt 12):1669–1674. 18033837 10.1099/jmm.0.47410-0

[44] Pisarello MLJ Vintiñi EO González SN. Decrease in lactobacilli in the intestinal microbiota of celiac children with a gluten-free diet, and selection of potentially probiotic strains. Can J Microbiol. 2014;61:32–37. 10.1139/cjm-2014-0472 25438612

[45] Nistal E Caminero A Vivas S. Differences in faecal bacteria populations and faecal bacteria metabolism in healthy adults and celiac disease patients. Biochimie. 2012;94:1724–1729. 22542995 10.1016/j.biochi.2012.03.025

[46] Hansen LBS Roager HM Søndertoft NB. A low-gluten diet induces changes in the intestinal microbiome of healthy Danish adults. Nat Commun. 2018;9:4630. 30425247 10.1038/s41467-018-07019-x PMC6234216

[47] De Filippis F Pasolli E Tett A. Distinct genetic and functional traits of human intestinal prevotella copri strains are associated with different habitual diets. Cell Host Microbe. 2019;25:444–453.e3. 30799264 10.1016/j.chom.2019.01.004

[48] Bonder MJ Tigchelaar EF Cai X. The influence of a short-term gluten-free diet on the human gut microbiome. Genome Med. 2016;8:45. 27102333 10.1186/s13073-016-0295-y PMC4841035

[49] Isaac DM Rajani S Yaskina M. Antitissue transglutaminase normalization postdiagnosis in children with celiac disease. J Pediatr Gastroenterol Nutr. 2017;65:195–199. 27906802 10.1097/MPG.0000000000001480

