# Supplementary figures and images for: Baseline Fecal Microbiota in Pediatric Patients With Celiac Disease Is Similar to Controls But Dissimilar After 1 Year on the Gluten-Free Diet
Source: JPGN Rep. 2021 Oct 13;2(4):e127. doi: 10.1097/PG9.0000000000000127 (PMC10191547; doi:10.1097/PG9.0000000000000127)

A

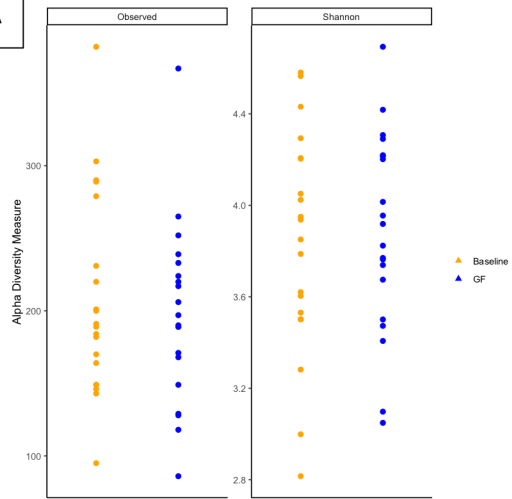

B

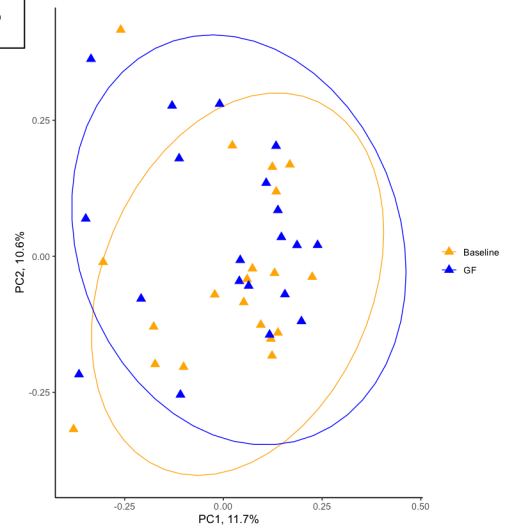

Supplement: Supplementary file 1 [file pg9-2-e127-s001.pdf]

A

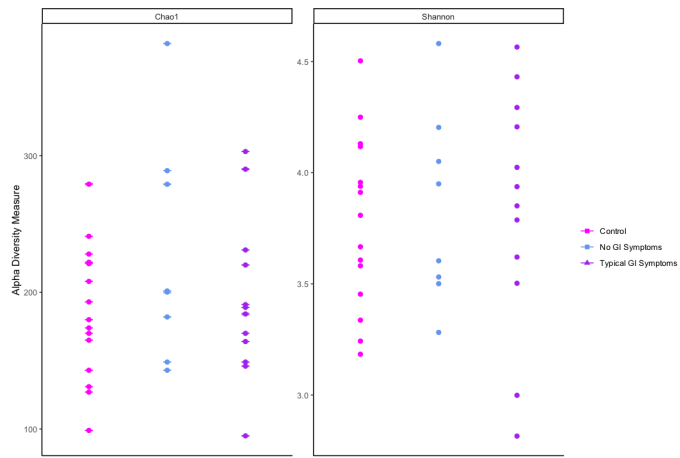

B

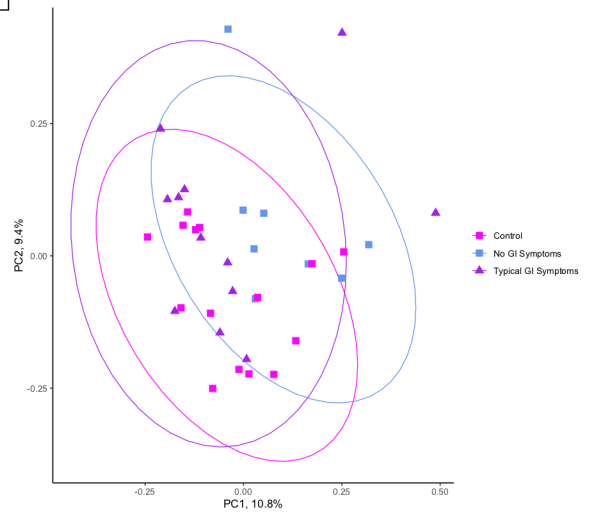

Supplement: Supplementary file 2 [file pg9-2-e127-s002.pdf]
